# A Comprehensive Review of the Physical Examination of the Elbow in the Throwing Athlete

**DOI:** 10.1002/atn2.70313

**Published:** 2026-10-29

**Authors:** Joshua R. Eskew, Bryan T. Soth, Jude Alawa, Todd M. McGrath, Tony Wanich, Carl M. Cirino, James B. Carr

**Affiliations:** ^1^ Hospital for Special Surgery Orthopedic Surgery New York New York U.S.A.

## Abstract

Elbow pain is a significant source of morbidity in overhead throwing athletes, arising from a spectrum of pathologies including ulnar collateral ligament injury, valgus extension overload, medial epicondylitis, ulnar neuritis, radiocapitellar pathology, and more. Accurate diagnosis requires a systematic physical examination combined with advanced imaging. This technical note describes a comprehensive examination protocol for the throwing athlete's elbow.

## Graphical abstract

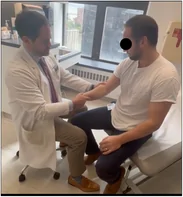

**VIDEO 1:** A comprehensive, stepwise display of the elbow physical examination in the throwing athlete is performed by the senior author. Video content can be viewed at https://doi.org/10.1002/atn2.70313.

Elbow pain is common in overhead throwing athletes and represents a significant source of morbidity, with the incidence of elbow injuries continuing to increase, particularly among adolescent baseball pitchers.^1^, ^2^ The differential diagnosis is broad and includes medial pathology such as ulnar collateral ligament (UCL) injury, medial epicondylitis, and ulnar neuritis; posterior pathology including valgus extension overload syndrome and olecranon stress injury; and lateral pathology such as radiocapitellar overload and osteochondritis dissecans of the capitellum. The overhead throwing motion subjects the elbow to nearly supraphysiological forces generating medial tensile stress, lateral compressive loading, and posterior shear, resulting in predictable injury patterns across all 3 compartments.^3^ Accurate diagnosis across this spectrum is critical for appropriate treatment planning and return‐to‐sport decision‐making.

The diagnostic evaluation of UCL injuries relies on a combination of careful history‐taking, thorough physical examination, and judicious use of advanced imaging.^4^ Athletes with elbow pathology typically present with pain that worsens during throwing activities, often accompanied by decreased throwing effectiveness with loss of control and/or velocity. The location of symptoms is an important diagnostic clue: medial pain during the acceleration phase suggests UCL or flexor‐pronator pathology; posteromedial pain during follow‐through raises concern for valgus extension overload; and lateral pain may indicate radiocapitellar or osteochondral pathology.^4^ The onset may be acute, associated with a popping or tearing sensation or gradual, with prodromal symptoms.^1^

Physical examination remains the cornerstone of elbow assessment in the throwing athlete. A comprehensive, compartment‐based examination is essential, as multiple pathologies frequently coexist. Provocative maneuvers including the static valgus stress test, modified milking maneuver, and moving valgus stress test are commonly employed to assess medial elbow stability.^3^ Dynamic valgus testing has been shown to be more reliable than static testing in assessing UCL tears.^1^ The moving valgus stress test is currently accepted as the gold standard clinical examination technique, with biomechanical studies showing that it produces significantly more UCL elongation than static stress testing, particularly during the extension phase near 90° of flexion.^5^

Although physical examination is essential, advanced imaging provides definitive diagnosis. Magnetic resonance imaging and magnetic resonance arthrography remain the gold standard for imaging evaluation, with magnetic resonance arthrography showing sensitivity ranging from 81% to 100% and specificity from 91% to 100%.^6^ However, stress ultrasonography has emerged as an increasingly valuable adjunct, offering dynamic assessment with sensitivity of 96% and specificity of 81% for UCL injury detection.^7^ The combined use of magnetic resonance arthrography and stress ultrasound has shown increase in diagnostic accuracy to 98%, superior to either modality alone.^8^

Despite the importance of physical examination in the diagnostic algorithm, published data characterizing the sensitivity and specificity of individual elbow examination maneuvers remain limited. This article presents a comprehensive examination technique for the throwing athlete's elbow, emphasizing a systematic, compartment‐based approach, and reviews the available evidence regarding diagnostic test characteristics for medial elbow instability.

## EXAMINATION TECHNIQUE

Prior to performing any specific examination maneuvers, a general assessment of the entire kinetic chain is essential. Because the throwing motion is a whole‐body activity, deficits at any segment can propagate forces to the elbow. The examiner should assess hip rotation and strength, core stability (via a 2‐leg and single‐leg squat), scapular positioning and dyskinesis, and shoulder motion including the total arc of rotation and internal rotation deficit, all of which may contribute to elbow pathology when deficient. After this kinetic chain assessment, focused examination of the upper extremity proceeds. The examination begins with inspection for asymmetry, swelling, muscle atrophy, and carrying‐angle variation. Palpation is then performed systematically along the medial, lateral, and posterior elbow to identify focal tenderness about the ulnar collateral ligament (UCL), flexor‐pronator mass, lateral epicondyle, radial tunnel, extensor tendon origin, radiocapitellar joint, and olecranon tip. Active and passive ranges of motion are assessed and compared with the contralateral limb, noting any loss of terminal extension or painful arcs suggestive of valgus extension overload. A comprehensive strength examination is then performed for elbow flexion, extension, supination and pronation, and also wrist and finger flexion and extension. After general inspection and palpation are complete, specific maneuvers are performed to localize pathology (Video 1). The following special tests are then performed.

### Static Valgus Stress Test

The elbow is placed in neutral and 30° to 45° of flexion. The examiner's hand is positioned on the lateral border of the elbow, and a valgus stress is applied while assessing for instability, apprehension, or discomfort (Figure 1).

**FIGURE 1:**
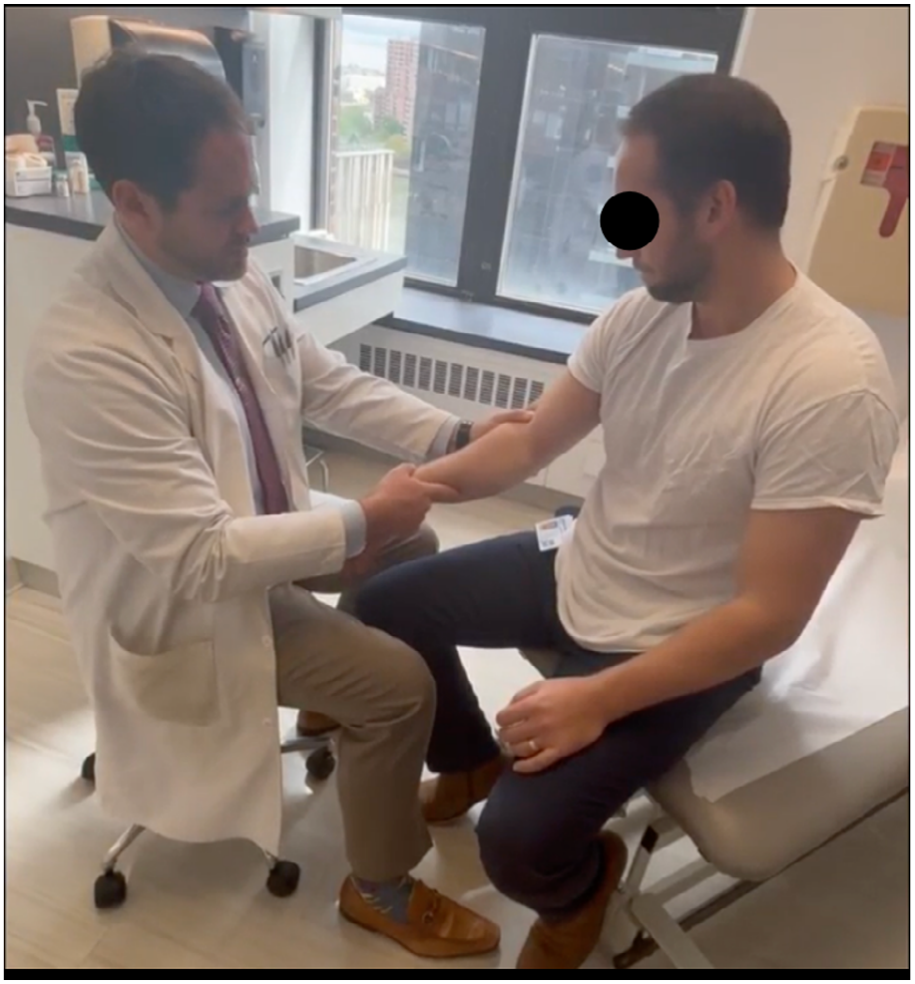
Static valgus stress test. The examiner assesses a patient's right elbow. The elbow is placed in neutral and 30°‐45° of flexion. The examiner's hand is positioned on the lateral border of the elbow, and a valgus stress is applied while assessing for instability, apprehension, or discomfort.

### Moving Valgus Stress Test

The examiner stands and positions the patient in the 90 to 90 position (shoulder abducted at 90° and elbow flexed at 90°). The upper extremity is brought into the layback or maximal external rotation position. The elbow is then gradually extended from 90° of flexion while maintaining a valgus stress. The “shear zone,” typically between 30° and 60° of flexion, is where maximal stress is applied to the UCL. The examiner assesses for apprehension, pain, or discomfort through the arc of motion (Figure 2).

**FIGURE 2:**
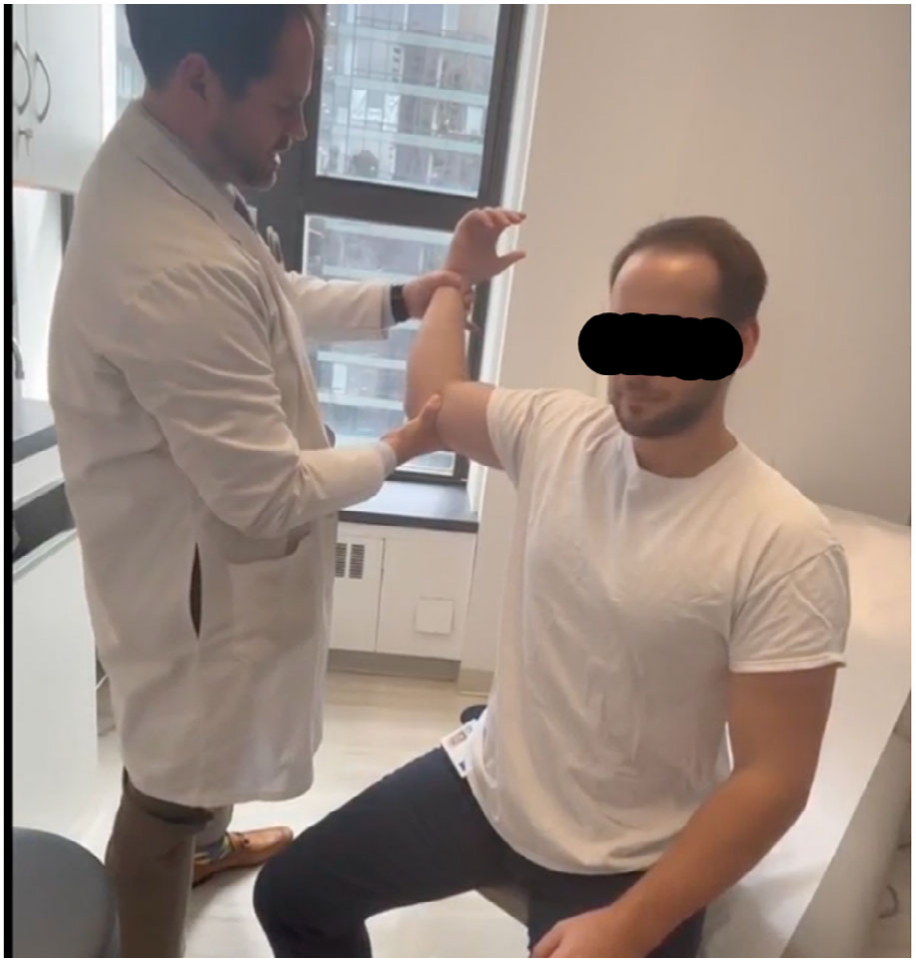
MVST. The examiner assesses a patient's right elbow. The examiner stands and positions the patient in the 90‐90 position (shoulder abducted at 90° and elbow flexed at 90°). The upper extremity is brought into the layback or maximal external rotation position. The elbow is then gradually extended from 90° of flexion while maintaining a valgus stress. The “shear zone,” typically between 30° and 60° of flexion, is where maximal stress is applied to the UCL. The examiner assesses for apprehension, pain, or discomfort through the arc of motion. (MVST, moving valgus stress test; UCL, ulnar collateral ligament.)

### Milking Maneuver

The shoulder is brought to 45° of flexion in the forward scapular plane. The elbow is placed into the 30° to 60° shearing zone, and a valgus stress is applied with one hand positioned under the elbow and the other at the wrist. This maneuver can be performed at various angles of elbow flexion‐extension (Figure 3).

**FIGURE 3:**
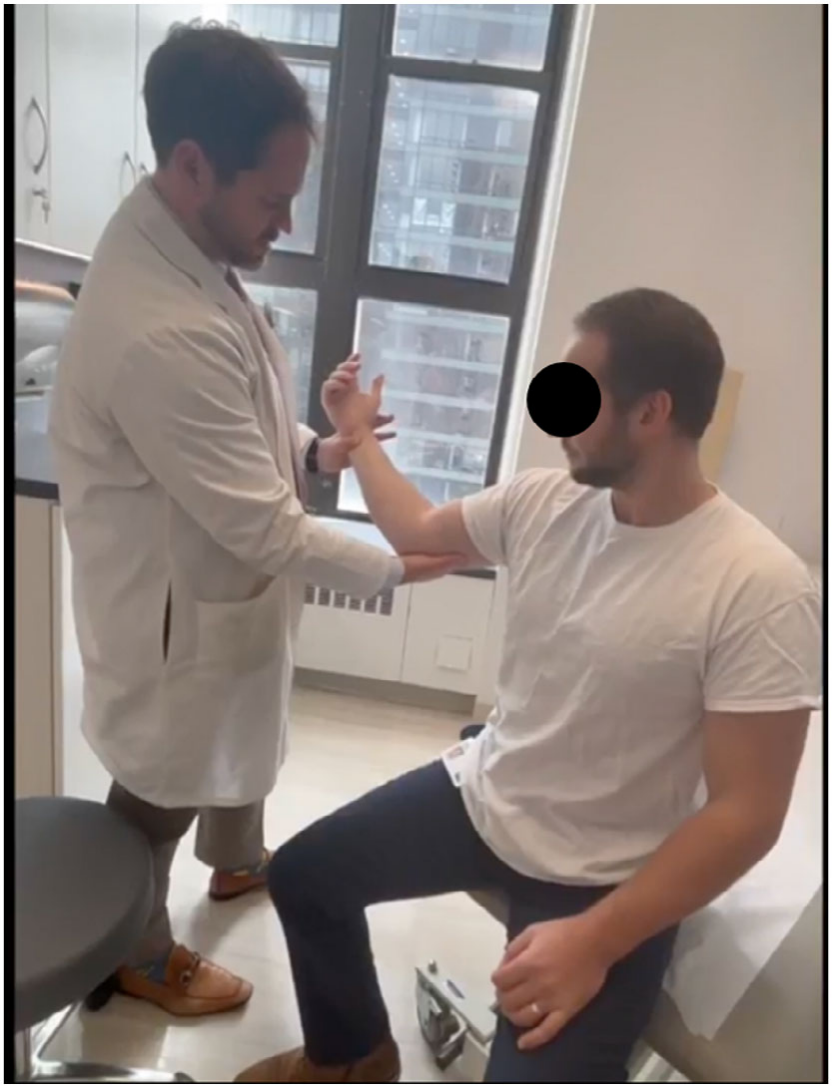
Milking maneuver. The examiner assesses a patient's right elbow. The shoulder is brought to 45° of flexion in the forward scapular plane. The elbow is placed into the 30°‐60° shearing zone, and a valgus stress is applied with one hand positioned under the elbow and the other at the wrist. This maneuver can be performed at various angles of elbow flexion‐extension.

### Repetitive Valgus Stress Test

The shoulder is brought into 45° of flexion in the scapular plane with the forearm in pronation. A repetitive valgus stress is applied to the elbow for 15 to 20 seconds. It is important to isolate the elbow so that shoulder motion does not occur concurrently during this maneuver. This test attempts to reproduce medial elbow symptoms by simulating the repetitive stress of throwing and can be especially helpful in patients with chronic UCL injury (Figure 4).

**FIGURE 4:**
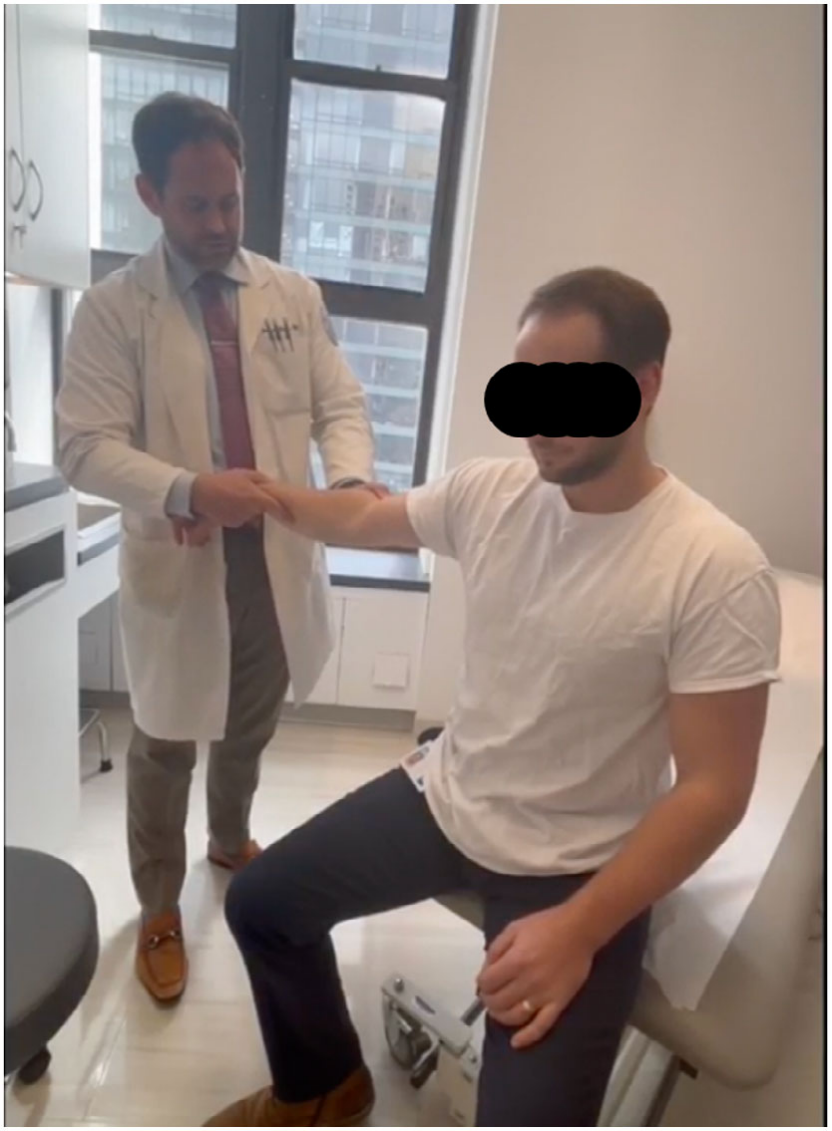
Repetitive valgus stress test. The examiner assesses a patient's right elbow. The shoulder is brought into 45° of flexion in the scapular plane with the forearm in pronation. A repetitive valgus stress is applied to the elbow for 15‐20 seconds. It is important to isolate the elbow so that shoulder motion does not occur concurrently during this maneuver. This test attempts to reproduce medial elbow symptoms by simulating the repetitive stress of throwing and can be especially helpful in patients with chronic UCL injury. (UCL, ulnar collateral ligament.)

### Assessment of the Cubital Tunnel and Ulnar Nerve

The shoulder is flexed to 90° in the scapular plane, and the elbow is maximally flexed. A combination of the elbow flexion compression test and resistance test is performed, along with a Tinel sign over the ulnar nerve, to assess for ulnar nerve irritation or symptoms.

### Assessment of Posterior Elbow Pathology: Posteromedial Impingement and Olecranon Stress Injuries

The elbow is brought into full flexion, and resisted extension is assessed. The bouncing elbow test is performed by repetitively bouncing the elbow into hyperextension to evaluate for posteromedial impingement (Figure 5).

**FIGURE 5:**
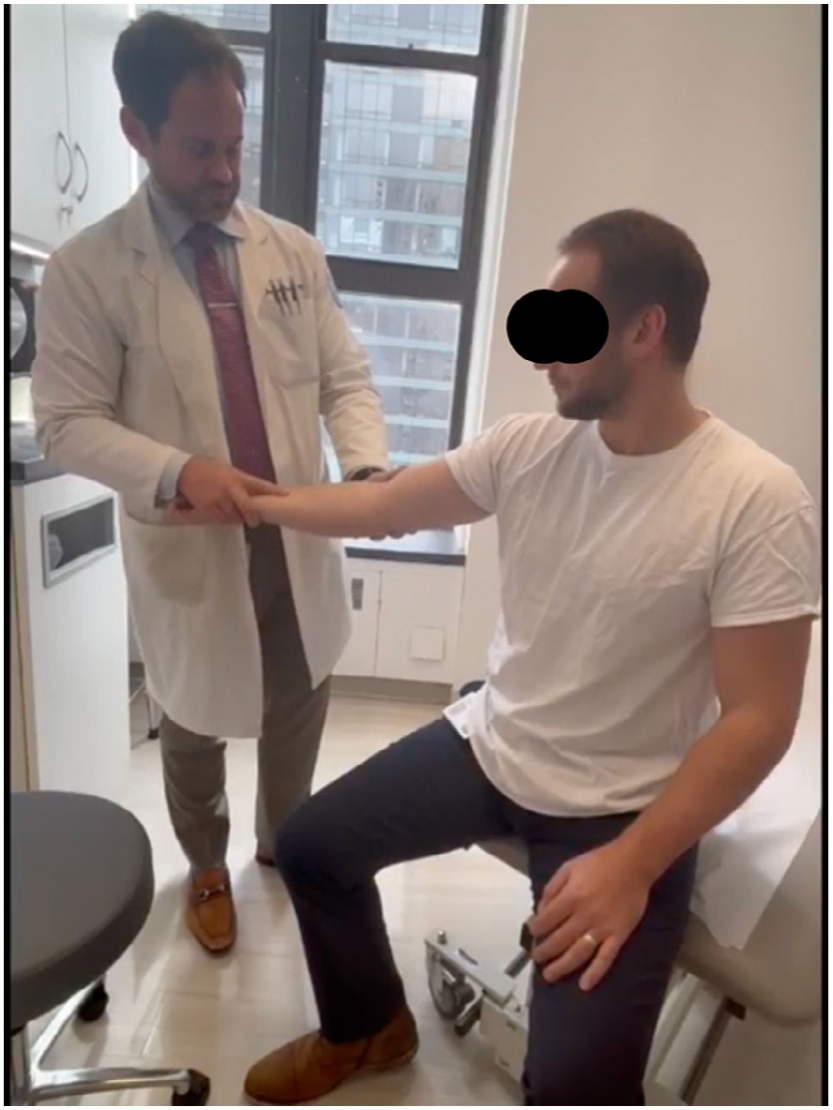
Assessment of posterior elbow pathology: posteromedial impingement and olecranon stress injuries. The examiner assesses a patient's right elbow. The elbow is brought into full flexion, and resisted extension is assessed. The bouncing elbow test is performed by repetitively bouncing the elbow into hyperextension to evaluate for posteromedial impingement.

## DISCUSSION

The physical examination of the throwing athlete's elbow requires a comprehensive, compartment‐based approach that integrates knowledge of elbow anatomy, throwing biomechanics, and the kinetic chain contributions to upper‐extremity loading. Pathology in overhead throwers commonly spans multiple compartments, and a systematic examination that evaluates the full spectrum of medial, posterior, and lateral elbow structures is essential for accurate diagnosis and treatment planning.^9^ Our technique emphasizes the importance of dynamic valgus stress testing, which has been shown to be superior to static examination methods.^1^

The moving valgus stress test (MVST) represents the most biomechanically validated examination technique for UCL assessment. Wigton et al. showed in a cadaveric study that the MVST produces significantly greater UCL elongation compared with static stress testing (*P* < .001), with maximum change in length occurring during extension near 90° of flexion.^5^ This biomechanical evidence supports the clinical use of the MVST as a superior examination technique for UCL injuries. The test is performed by applying valgus stress to the elbow while moving it through a range of motion from full flexion to approximately 30° of flexion, with reproduction of the patient's pain indicating a positive test.^9^

The milking maneuver, performed with the elbow flexed beyond 90°, and the thumb pulled laterally to create valgus stress, represents another dynamic assessment technique.^3^ Although consensus guidelines recommend dynamic valgus testing, including the milking maneuver, as more reliable than static testing, specific sensitivity and specificity data for this individual maneuver are not well established in the literature.^1^ DeMoss et al. evaluated ultrasound‐assisted assessment during the milking maneuver and found that it produced measurable changes in the medial joint space width, although slightly less than the valgus stress test positions.^10^

The traditional valgus stress test, performed at 30° of elbow flexion with the forearm supinated, has been evaluated primarily in the context of stress imaging rather than as an isolated clinical examination finding. When combined with stress ultrasound, valgus stress testing at 30° of flexion shows a sensitivity of 88.1% and specificity of 61.5% for complete UCL rupture using a cutoff value of 0.5 mm of joint gapping.^11^ At 90° of flexion, the sensitivity decreases to 81.0% with specificity of 66.4% using a 1.0‐mm cutoff.^11^ The examination maneuvers with detailed specificity and sensitivity are detailed in Table 1.

**TABLE 1:** Physical Examination Maneuvers for Ulnar Collateral Ligament Injury Assessment

**Examination Maneuver**	**Technique**	**Positive Finding**	**Sensitivity**	**Specificity**
MVST	Apply valgus torque to the elbow while moving from full flexion to approximately 30° of extension with the shoulder abducted to 90°	Reproduction of medial elbow pain within the “shear zone” between 120° and 70° of flexion, maximal at 90°	100%	75%
Milking maneuver	With elbow flexed beyond 90° and forearm supinated, pull the thumb laterally to create valgus stress on the elbow	Medial elbow pain or apprehension with subjective sense of instability	100%	75%
Static valgus stress test	Stabilize the humerus with elbow flexed to 30° and forearm supinated; apply valgus force to the elbow	Increased medial joint opening, pain, or laxity compared with contralateral side	65%	50%

MVST, moving valgus stress test.

It is important to note that the available diagnostic accuracy data predominantly reflect imaging‐assisted examination (stress ultrasound or stress radiography) rather than clinical examination alone. Roedl et al. showed that stress ultrasound with joint gapping greater than 1.0 mm compared with the contralateral elbow yielded sensitivity of 96%, specificity of 81%, and accuracy of 87% for diagnosing UCL tears.^8^ The arthroscopic capsular release Appropriateness Criteria cite similar values for dynamic stress ultrasound, with sensitivity of 96% and specificity of 81%.^7^

The diagnostic performance of clinical examination alone, without imaging assistance, remains less well characterized. A systematic review and meta‐analysis by Rashidi et al. examining UCL tears of the thumb found that clinical examination showed pooled sensitivity of 97% (95% confidence interval, 93%‐99%) for ruling out UCL tears, but specificity of only 85% (95% confidence interval, 78%‐91%) for ruling in tears.^12^ Although this study focuses on thumb UCL injuries rather than elbow UCL injuries, it highlights the general principle that clinical examination is highly sensitive but less specific for UCL pathology (Table 2).

**TABLE 2:** Pearls and Pitfalls of the Throwing Elbow Examination

**Pearls**	**Pitfalls**
Assess the entire kinetic chain before focusing on the elbow: deficits in hip rotation, core strength, and shoulder motion (especially internal rotation deficit) frequently contribute to elbow overload.	Do not focus exclusively on the UCL; multiple pathologies commonly coexist (e.g., UCL insufficiency with valgus extension overload and ulnar neuritis).
The MVST is most sensitive when performed with the shoulder in maximum external rotation (layback position), reproducing the late‐cocking phase of throwing.	Pain with the MVST outside the shear zone (30°‐120° of flexion) is unlikely to represent UCL pathology and consider other diagnoses such as medial epicondylitis or cubital tunnel syndrome.
Compare range of motion bilaterally; a flexion contracture of 5°‐10° is common in competitive throwers and represents an adaptive change rather than pathology in isolation.	Avoid interpreting bilateral valgus laxity on the static valgus stress test as pathologic; many high‐level throwers show asymptomatic laxity; a comparison with the contralateral side and symptom correlation is essential.
Palpate the UCL directly (2 cm distal to the medial epicondyle); point tenderness at this location is 81%‐94% sensitive for UCL pathology and helps localize injury vs flexor‐pronator mass pathology.	Patient guarding can produce false‐negative results; examining the contralateral limb first and applying stress slowly may improve accuracy.
Always examine for ulnar nerve involvement; ulnar neuritis or subluxation coexists with UCL pathology in a significant proportion of throwers and may alter management.	In skeletally immature throwers, do not assume UCL injury; medial epicondyle apophysitis (“Little Leaguer's elbow”), apophyseal fractures, and osteochondritis dissecans of the capitellum must be considered.
The repetitive valgus stress test is particularly useful in chronic or partial UCL injuries; reproducing symptoms over 15‐20 s can unmask subtle insufficiency.	A negative examination does not exclude UCL pathology; advanced imaging (MRA or stress ultrasound) should be obtained when clinical suspicion remains high.

MRA, magnetic resonance arthrography; MVST, moving valgus stress test; s, seconds; UCL, ulnar collateral ligament.

The integration of physical examination findings with advanced imaging remains essential for accurate diagnosis. Magnetic resonance imaging and magnetic resonance arthrography (MRA) provide the best combination of sensitivity and specificity for UCL evaluation, with MRA showing superior performance.^6^ However, the portability, cost‐effectiveness, and dynamic capabilities of stress ultrasound make it an increasingly valuable tool, particularly when combined with MRA.^8^, ^13^ Recent consensus statements emphasize that advanced imaging in the form of magnetic resonance imaging or MRA should be performed in patients with suspected UCL tears who plan to continue overhead sport participation.^2^

Several limitations exist in the current literature regarding UCL examination techniques. First, there are a lack of high‐quality studies directly comparing the sensitivity and specificity of individual physical examination maneuvers (milking maneuver, MVST, and valgus stress test) using surgical findings as the gold standard. Most available data combine clinical examination with imaging modalities, making it difficult to isolate the diagnostic performance of examination alone. Second, the diagnostic accuracy of physical examination may vary based on examiner experience, patient guarding, and the chronicity of injury. Third, partial UCL tears present a particular diagnostic challenge, as they may not show the same degree of laxity as complete tears.^11^, ^14^

Future research should focus on prospective studies evaluating the diagnostic test characteristics of individual UCL examination maneuvers compared with surgical findings as the reference standard. Additionally, investigation into the inter‐rater and intra‐rater reliability of these examination techniques would provide valuable information for clinical practice. The role of stress ultrasound as a point‐of‐care diagnostic tool deserves further study, particularly in comparison with MRA.

In conclusion, the physical examination of the throwing elbow requires a comprehensive approach incorporating multiple dynamic valgus stress maneuvers. Although the MVST is biomechanically validated as the superior clinical examination technique, the specific sensitivity and specificity of individual examination maneuvers remain incompletely characterized in the literature. Dynamic stress ultrasound shows excellent sensitivity (96%) and good specificity (81%) for UCL injury detection and represents a valuable adjunct to clinical examination. MRI and MRA remain the gold standard for definitive diagnosis, with MRA offering the best combination of sensitivity (81%‐100%) and specificity (91%‐100%). A systematic examination approach combined with appropriate advanced imaging enables accurate diagnosis and optimal treatment planning for overhead athletes with UCL injuries.

## DISCLOSURES

The authors (J.R.E., B.T.S., J.A., T.M.M., T.W., C.M.C., J.B.C.) declare that they have no known competing financial interests or personal relationships that could have appeared to influence the work reported in this paper.

## References

[1] Herring SA , Kibler WB , Putukian M , et al. Initial assessment and management of select musculoskeletal injuries: A team physician consensus statement. Med Sci Sports Exerc. 2024;56:385‐401. 10.1249/MSS.0000000000003324 37847756

[2] Erickson BJ , Hurley ET , Mojica ES , Jazrawi LM . Elbow ulnar collateral ligament tears: A modified consensus statement. Arthroscopy. 2023;39:1161‐1171. 10.1016/j.arthro.2022.12.033 36796550

[3] Hariri S , Safran MR . Ulnar collateral ligament injury in the overhead athlete. Clin Sports Med. 2010;29:619‐644. 10.1016/j.csm.2010.06.007 20883901

[4] Ciccotti MC , Ciccotti MG . Ulnar collateral ligament evaluation and diagnostics. Clin Sports Med. 2020;39:503‐522. 10.1016/j.csm.2020.02.002 32446571

[5] Wigton MD , Schimoler PJ , Kharlamov A , et al. The moving valgus stress test produces more ulnar collateral ligament change in length during extension than during flexion: A biomechanical study. J Shoulder Elbow Surg. 2020;29:1230‐1235. 10.1016/j.jse.2019.12.025 32197808

[6] Campbell RE , McGhee AN , Freedman KB , Tjoumakaris FP . Diagnostic imaging of ulnar collateral ligament injury: A systematic review. Am J Sports Med. 2020;48:2819‐2827. 10.1177/0363546520937302 32735456

[7] Chen KC , Ha AS , Bartolotta RJ , et al. ACR appropriateness criteria acute elbow and forearm pain. J Am Coll Radiol. 2024;21(suppl 11):S355‐S363. 10.1016/j.jacr.2024.08.012 39488347

[8] Roedl JB , Gonzalez FM , Zoga AC , et al. Potential utility of a combined approach with US and MR arthrography to image medial elbow pain in baseball players. Radiology. 2016;279:827‐837. 10.1148/radiol.2015151256 27183408

[9] Bi AS , Jazrawi LM , Cohen SB , Erickson BJ . The physical examination of the throwing elbow. Clin Sports Med. 2025;44:129‐142. 10.1016/j.csm.2024.05.003 40021248

[10] DeMoss A , Millard N , McIlvain G , et al. Ultrasound‐assisted assessment of medial elbow stability. J Ultrasound Med. 2018;37:2769‐2775. 10.1002/jum.14631 29655251

[11] Park JY , Kim H , Lee JH , et al. Valgus stress ultrasound for medial ulnar collateral ligament injuries in athletes: Is ultrasound alone enough for diagnosis? J Shoulder Elbow Surg. 2020;29:578‐586. 10.1016/j.jse.2019.12.005 32067711

[12] Rashidi A , Haj‐Mirzaian A , Dalili D , Fritz B , Fritz J . Evidence‐based use of clinical examination, ultrasonography, and MRI for diagnosing ulnar collateral ligament tears of the metacarpophalangeal joint of the thumb: Systematic review and meta‐analysis. Eur Radiol. 2021;31:5699‐5712. 10.1007/s00330-020-07666-z 33459856

[13] Hultman KL , Goldman BH , Nazarian LN , Ciccotti MG . Ultrasound examination techniques for elbow injuries in overhead athletes. J Am Acad Orthop Surg. 2021;29:227‐234. 10.5435/JAAOS-D-20-00935 33273401

[14] Ciccotti MC , Paul RW , Gawel RJ , et al. In vivo effect of partial ulnar collateral ligament tear location on ulnohumeral joint gapping with stress ultrasound in baseball pitchers. Am J Sports Med. 2023;51:3280‐3287. 10.1177/03635465231197627 37715521

